# Major Histocompatibility Complex (MHC) Markers in Conservation Biology

**DOI:** 10.3390/ijms12085168

**Published:** 2011-08-15

**Authors:** Beata Ujvari, Katherine Belov

**Affiliations:** Faculty of Veterinary Science, University of Sydney, RMC Gunn Bldg, Sydney, NSW 2006, Australia; E-Mail: beata.ujvari@sydney.edu.au

**Keywords:** Major Histocompatibility Complex (MHC), conservation biology, genetic rescue, captive breeding, Tasmanian devil (*Sarcophilus harrisii*), Devil Facial Tumor Disease (DFTD), next-generation sequencing

## Abstract

Human impacts through habitat destruction, introduction of invasive species and climate change are increasing the number of species threatened with extinction. Decreases in population size simultaneously lead to reductions in genetic diversity, ultimately reducing the ability of populations to adapt to a changing environment. In this way, loss of genetic polymorphism is linked with extinction risk. Recent advances in sequencing technologies mean that obtaining measures of genetic diversity at functionally important genes is within reach for conservation programs. A key region of the genome that should be targeted for population genetic studies is the Major Histocompatibility Complex (MHC). MHC genes, found in all jawed vertebrates, are the most polymorphic genes in vertebrate genomes. They play key roles in immune function via immune-recognition and -surveillance and host-parasite interaction. Therefore, measuring levels of polymorphism at these genes can provide indirect measures of the immunological fitness of populations. The MHC has also been linked with mate-choice and pregnancy outcomes and has application for improving mating success in captive breeding programs. The recent discovery that genetic diversity at MHC genes may protect against the spread of contagious cancers provides an added impetus for managing and protecting MHC diversity in wild populations. Here we review the field and focus on the successful applications of MHC-typing for conservation management. We emphasize the importance of using MHC markers when planning and executing wildlife rescue and conservation programs but stress that this should not be done to the detriment of genome-wide diversity.

## 1. Introduction

Ever since the development of protein electrophoresis in the 1970s, biologists have realized that most natural populations exhibit high levels of genetic diversity [1]. Genetic diversity is the base material for selective processes. High levels of diversity enable populations to respond to threats such as pathogens, predators, and to long term effects such as environmental change [2]. Conversely, low levels of genetic diversity may limit a population’s ability to respond to these threats in both the long and short term [3]. The level of genetic diversity within a population represents a balance between gene flow, mutation, drift (random changes in allele frequencies), and natural selection. Habitat fragmentation can result in decreased effective population size and concurrent increase in the rate of inbreeding. The diminishing gene flow among fragmented populations may further exacerbate the loss of polymorphism. Genetic diversity is generated by mutation, and in small populations it may be eroded by drift. Natural selection may either reduce genetic diversity by fixation of alleles or promote diversity as a result of balancing or diversifying selection [4].

Genetic diversity may be reduced as a consequence of periods of fragmentation and decreased population size (bottlenecks). At first, it may seem that such loss of genetic diversity is only of concern for long-term evolutionary adaptation. However, there are immediate short-term implications as well. Loss of genetic diversity is intimately related to an increased risk of inbreeding depression resulting in decreased growth rate, fertility, fecundity and offspring viability [5–12]. Although the negative effects of inbreeding may be reduced, or purged, by selection against deleterious alleles, it is highly unlikely to completely eliminate its impact on organismal fitness [2,13]. Populations that have lost genetic diversity may also suffer from an increased probability of extinction as a consequence of increased vulnerability to novel pathogens [14,15]. Hence, the maintenance of genetic diversity is of fundamental importance in conservation biology [4,6–8,11,16–21].

During the last two decades, microsatellites (sections of DNA consisting of very short repeated nucleotide sequences) have frequently been employed in quantifying population genetic diversity and the results from such studies have often provided the basis for management recommendations (reviewed in [22]). The frequent use of microsatellites in conservation genetics is commonly based on the assumption that these markers are neutral *i.e.*, not directly targeted by selection. However, emerging evidence shows that patterns of variation and divergence in adaptive traits are not always associated with concomitant variation in neutral markers and several studies have questioned the validity of using only neutral markers for development of conservation strategies [22–30].

Two central questions in conservation genetics are: (1) the degree to which genetic bottlenecks and low effective population size will reduce genetic diversity within a population; and (2) the impact of this reduction on the population’s long-term viability. In particular, will genetic diversity be reduced to a similar degree throughout the genome, or will some loci be affected more than others? The strength of the relationship between genetic variation and effective population size varies for different categories of markers which are subject to different intensities of selection [4,21,22,31]. Selection is likely to retain higher levels of genetic diversity at some functionally important loci, despite reductions in variation at other parts of the genome. Therefore, the use of genetic markers linked to adaptive traits, including genes involved in immune defense, reproduction and some physiological functions, is important [32,33]. Recent studies suggest that the Major Histocompatibility Complex (MHC) loci are particularly suited to this role [7,11,21,31,34–40]. These studies are reviewed below.

## 2. Results and Discussion

### 2.1. An Overview of the Major Histocompatibility Complex

The Major Histocompatibility Complex (MHC) plays a crucial role in the vertebrate immune system by encoding a collection of immune and non-immune related molecules [41,42]. The term MHC was derived from early transplant studies in humans and mice that revealed the role of glycoproteins encoded by MHC in self-identification (or histocompatibility) [43,44]. In 1975, Doherty and Zinkernagel linked the role of the MHC molecules to antigen presentation [45,46]. Since then MHC class I and class II loci have been shown to exhibit an extraordinarily high degree of polymorphism and over 1000 HLA-A, HLA-B and HLA-C MHC Class I molecules as well as hundreds of DRB alleles of Class II loci have been characterized in human populations [47]. Based on their structure and function MHC genes generally cluster into three groups, called Class I, II and III. The main function of the ubiquitously expressed classical Class Ia molecules is to present foreign cytosolic peptides to CD8+ cytotoxic T cells [48,49]. Non-classical MHC Class I molecules (Ib) accomplish a variety of cellular tasks commonly performed by epithelial cells, specifically in areas of cellular transport and regulation of lymphocyte responses to altered epithelial cells and possibly bacterial antigens [50]. In humans, MHC Class II molecules are only expressed on the surface of professional antigen presenting cells, such as macrophages, dendritic- and B-cells [51]. In dogs and some other species, they are expressed on both B- and T-cells [52]. Class II molecules present exogenously derived antigens to CD4+ T helper cells triggering an immune response, such as activation of antibody production by B-cells, resulting in the destruction of the invaded cell [51]. The MHC Class II molecules are also classified into classical (IIa) and non-classical (IIb) categories, respectively, based on their ability or inability to present antigens.

MHC Class III contains a variety of genes that do not have antigen presenting capacity, but code for other immune functions, such as complement components (e.g., C2, C4, factor B) and cytokines (e.g., TNF-α [53]).

### 2.2. Evolution of MHC Polymorphism

Two, not mutually exclusive, hypotheses have been suggested to explain the high level of MHC polymorphism: (i) pathogen-driven selection [54–57]; and (ii) MHC-based mate choice [58–60]. Given the central role of MHC in the vertebrate immune system, the pathogen-driven selection may be a more likely candidate for explaining the high MHC diversity observed in most vertebrates, and may serve as the underlying reason for MHC-based mate choice.

It is generally believed that some form of pathogen-driven balancing selection, a broad term that identifies any kind of natural selection where no single allele is absolutely most fit, is responsible for the high polymorphism of the MHC genes, but the exact nature of the selection continues to be a topic of debate [37,61]. A recent study, however, shows that different modes of MHC selection are operating in different systems and during different times, suggesting that the mechanisms for maintenance of MHC polymorphism in natural populations are likely to be far more complex than previously envisioned [37].

### 2.3. Quantifying MHC Diversity

The primary use of MHC genes in conservation to date has been for quantifying genetic diversity of natural populations, without specific conservation management implications (Table 1). The extraordinary polymorphism of MHC genes observed in vertebrates [62] prompted biologists to focus on the most variable regions of MHC molecules, the peptide binding region (PBR) of either the MHC Class I or Class II molecules. Most of the allelic variation in the peptide-binding regions is maintained by selection processes, but MHC diversity is also generated through gene duplications and copy number variation [63]. Due to the complex genomic organization and high sequence variation of MHC loci, accurate genotyping of MHC variation can prove to be rather challenging and cumbersome. Several assays including mixed lymphocyte response assay (MLR), PCR and non-PCR based molecular methods have been developed to measure MHC polymorphism between individuals and within populations. The most frequently used techniques, such as Restriction Fragment Length Polymorphism (RFLP), Single Strand Conformation Polymorphism (SSCP), Denaturing Gradient Gel Electrophoresis (DGGE), Reference Strand-mediated Conformational Analysis (RSCA) and cloning followed by sequence-based typing of PCR products, have recently been reviewed in detail [64], we therefore do not expand further on the use of these methods. Instead we will briefly review the use of the most recently developed Next-Generation Sequencing (NGS) technologies. The rapid progress of high-throughput sequencing technologies has facilitated the development of so-called “-omics” (genomics, transcriptomics, metagenomics and proteomics) and revolutionized the scale and dimensions of accessible molecular information for evolutionary and conservation biology studies. Given the increasing capacity and speed of genome sequencing, and the shrinking cost of high-throughput sequencing, hundreds of vertebrate and invertebrate genomes and transcriptomes have been sequenced (reviewed in [65], c.f. GOLD, the Genomes OnLine Database v 3.0 [66]). The genome of the endangered Giant panda (*Ailuropoda melanoleuc*) has recently been sequenced and the number of endangered species targeted for genome sequencing is rapidly increasing [67]. The ability to use genomic sequences from closely related species also helps with design of genetic markers in endangered species [65,68,69]. Additionally, the genomes of thousands of pathogens and microorganisms have been sequenced, allowing the study of the co-evolutionary arms race of hosts and parasites, and the selection forces driving species extinctions (e.g., Amphibian Chytridiomycosis [70]). A key factor in conservation is to understand the spatiotemporal changes in host resistance to pathogens in natural populations, particularly for populations at high risk of disease outbreaks or pathogen introductions, such as in the Tasmanian devil (*Sarcophilus harrisii*) [71]. The use of NGS technology will enable conservation biologists to elucidate the immunogenetic status of small or endangered populations, and hence facilitate appropriate risk assessments and design management strategies.

The rapid evolution of NGS technologies will enable such a multi-gene approach. The latest ultra high-throughput Next-Generation Sequencing (NGS) technologies are relatively low cost, quick and easy to scale up or down (reviewed in [72,73]). A few studies on non-model animals already exist using these latest technologies to characterize and quantify MHC polymorphism in various species, for example in bank voles (*Myodes glareolus*) [74] and the collared flycatcher (*Ficedula albicollis*) [75], and it will not be long before next-generation sequencing becomes the accepted tool in conservation genetics [76]. A software package has been developed to assist in the analysis of next-generation data to identify multilocus gene families [75,77,78]. It is clear that NGS will facilitate the real-time monitoring of microevolutionary processes of host-parasite interactions and the co-analysis of genotypic and phenotypic evolutionary processes on a multigene level.

### 2.4. MHC in Conservation Biology

Maintenance of high levels of MHC polymorphism is crucial to counteract novel pathogenic challenges and to ensure organismal long-term survival [36,63,79–81]. In spite of its unambiguous fitness significance, a dispute between Hughes [32] and peers in the early ‘90s highlighted a major apprehension about the sole use of MHC markers in conservation genetics. Opponents argued that maximizing allele diversity at MHC loci would lead to the loss of genetic diversity at many other, equally important loci [82–84]. Acevedo-Whitehouse and Cunningham [85] recently suggested a broader approach by incorporating other candidate immune genes to understand wildlife immunogenetics. We support this notion and suggest that conservation programs should take into account as many genetic markers as possible, including MHC genes.

As mentioned previously, MHC markers have been used on endangered species (a selection of these studies is summarized in Table 1) [7,38–40,86–90]. MHC genes have been shown to be associated with individual variation in parasite load [57,91] local adaptations [92], maternal-foetal interactions [93,94] and life-time reproductive success [95]. Individual variation in MHC genes has been shown to be a major component in mate choice [96–98] by providing offspring with an optimal MHC repertoire [98,99]. MHC genes have also been used to plan captive breeding programs [24,38–40,86,88–109] (Table 1). We argue that MHC typing has an important place in conservation genetics, and should be used alongside other measures of genetic variability.

### 2.5. The Role of MHC in Captive Breeding

In order to minimize kinship, and reduce the deleterious effects of inbreeding in captive breeding programs, zoos rely on studbooks [110–112]. Studbooks have been employed successfully in many species. In 2009, the World Association of Zoos and Aquariums counted 118 active international studbooks, including 159 species and/or sub-species [113], including the red panda (*Ailurus fulgens*) [114], okapi (*Okapia johnstoni*) and the lowland gorilla (*Gorilla gorilla gorilla*) [110]. Captive management could benefit from the addition of genetic management, including MHC data, to the studbook process [112]. By measuring MHC diversity in captive populations, zoo staff would be forewarned about the resilience of the population to pathogen challenges. Populations with low MHC diversity should be managed with caution, and additional MHC alleles introduced into the population if at all possible.

### 2.6. The Role of MHC in Genetic Rescue Programs

Translocation of individuals from genetically and demographically healthy population to populations suffering from significantly reduced genetic diversity (reviewed in [115,116]) allows genetic rescue. Several recent studies have shown that inbred populations can be ‘rescued’ by the introduction of migrants, either naturally [117], or as part of a management program [7,8,12,118–120]. Only one study so far has monitored MHC during a genetic rescue program. Madsen *et al.* [7] showed that the introduction of new genes into a severely inbred and isolated population of Swedish adders (*Vipera berus*) halted the population’s decline. The genetic polymorphism of MHC genes in the population increased following the introduction of new snakes. The once severely inbred and isolated population of Swedish vipers continues to thrive and expand [8].

### 2.7. The Role of MHC in Transmissible Cancer

The emergence of virus-associated, carcinogen-related wildlife cancers [121] and transmissible tumors [71] raises novel and important conservation concerns. Cancers can directly or indirectly affect conservation outcomes by severely reducing individual fitness, ultimately resulting in altered population dynamics and population declines. The existence of two naturally occurring clonally transmissible cancers, Tasmanian Devil Facial Tumor Disease (DFTD) and Canine Transmissible Venereal Tumor (CTVT) further highlights the importance of MHC variation in conservation biology. Both of these diseases are transmitted by physical contact. CTVT is a sexually transmitted tumor of canines, while DFTD affects the largest marsupial carnivore, the Tasmanian devil (reviewed in [71]). Both cancers are believed to have emerged and spread due to genetic bottlenecks and low MHC diversity in dog and devil populations [71,107,122]. Siddle *et al.* [107,122] found that the rapid spread of DFTD and decline in devil populations by over 80% was due to a lack of MHC Class I diversity in inbred devils [71]. Devils in the infected areas have functionally identical MHC genes which they share with DFTD cells [107,108]. Consequently, the devils’ immune system does not recognize the DFTD cells as non-self and hence does not mount an immune response. The canine disease is also believed to have emerged in inbred wolf populations with low MHC diversity, and then spread to MHC-disparate hosts when the tumor evolved the ability to evade the host immune response [71]. A third transmissible cancer has been observed in inbred populations of captive-bred golden hamsters [123,124] further emphasizing that MHC diversity not only increases the immunological fitness of populations by providing protection against pathogens, but also helps to shield individuals from transmissible cancers [71,125–127].

DFTD provides a powerful example of how the loss of genetic diversity within populations, together with an infectious disease with frequency-dependent transmission, can cause extinction and presents a cautionary tale and a warning for conservation biologists to be aware of unusual diseases in inbred populations [127]. In conclusion, we emphasize, that maintenance of maximal genetic diversity across the genome should be the ultimate goal in conservation.

## 3. Perspectives

Anthropogenic activities have resulted in the extinction of numerous species and massive reductions in the population numbers of others. A consequence of this is loss of genetic diversity and a primary focus of conservation biologists has been quantifying genetic diversity of endangered and threatened species. A wide range of different genetic markers have been employed in conservation studies. We argue that with increasing accessibility to next-generation sequencing technologies, MHC and other immune-related genes should be used in addition to other markers, to provide indirect measures of the immunological fitness of populations as well as the evolutionary and adaptive potential of populations—especially those threatened by disease. We emphasize that there is still scope to increase the use of MHC and other adaptive markers for management of captive-bred populations and for genetic rescue programs. Both of these conservation measures require understanding of complex evolutionary, genetic and non-genetic (environmental, behavioral and demographic) factors, and therefore it is crucial to monitor genetic diversity pre- and post-management. Future studies should also focus on the spatiotemporal changes in host resistance to pathogens in natural populations. We envisage that NGS technologies will soon become the main tool for conservation geneticists, and will enable the real-time monitoring of microevolutionary processes, including host-parasite evolution across populations and entire species.

## Figures and Tables

**Table 1.** A selection of studies using MHC markers in conservation biology.

**Species**	**Purpose of using MHC**	**Reference**
**Fish**		
Chinook salmon (*Oncorhynchus tshawytscha*)	Understanding local adaptations	[92]
Brown trout (*Salmo trutta*)	Quantifying genetic diversity, disease susceptibility and human impact	[104]
Gila trout (*Oncorhynchus gilae gilae*)	Quantifying genetic diversity	[105]
Guppy (*Poecilia reticulate*)	Comparison of different conservation breeding regimes	[128]
**Birds**		
Chatham Island black robin (*Petroica traversi*)	Monitoring genetic variation following bottleneck	[129]
Crested ibis (*Nipponia nippon*)	Quantifying genetic diversity and implications for reintroduction	[90]
Galapagos penguin (*Spheniscus mendiculus*)	Quantifying genetic diversity	[130]
Gouldian finch *(Erythrura gouldiae)*	Quantifying genetic diversity	[131]
Great reed warbler (*Acrocephalus arundinaceus*)	Comparison of genetic polymorphism of an outbred and an inbred species	[132]
Seychelles warbler (*Acrocephalus sechellensis*)		
Sonoran topminnow (*Poeciliopsis occidentalis*)	Identify units for conservation	[133]
Various birds of prey (for detailed list see references)	Various conservation applications	[101,134]
**Reptiles**		
European adder (*Vipera berus*)	Genetic rescue, monitoring the effect of translocation	[6,7]
Hungarian meadow viper (*Viper ursinii rakosiensis*)	Quantifying genetic diversity and level of inbreeding	[11]
Sand lizard (*Lacerta agilis*)	Quantifying the correlation between population size and genetic diversity	[22]
Tuatara (*Sphenodon punctatus*)	Quantifying genetic diversity	[38,39]
**Eutherian mammals**		
African elephant (*Loxodonta africana*)	Quantifying genetic diversity	[135]
Asian elephant (*Elephas maximus*)		
African green monkey (*Chlorocebus sabaeus*)	Quantifying gene expression	[136]
African wild dog (*Lycaon pictus*)	Quantifying genetic diversity	[137]
American bison (*Bison bison*)	Quantifying genetic diversity and resistance to malignant catarrhal fever	[138,139]
Australian bush rat (*Rattus fuscipes*)	Quantifying genetic diversity	[140]
Aye-aye (*Daubentonia madagascariensi*s)	Quantifying genetic diversity	[141]
Baiji the Chinese river dolphin (*Lipotes vexillifer*)	Quantifying genetic diversity	[142]
Bengal tiger (*Panthera tigris tigris*)	Quantifying genetic diversity	[106]
Brown bear (*Ursus arctos*)	Quantifying genetic diversity	[143]
California sea lion (*Zalophus californianus*)	Quantifying genetic diversity and susceptibility to urogenital cancer	[144,145]
California sea otter (*Enhydra lutris nereis*)	Quantifying genetic diversity and bottleneck	[100]
Cheetah (*Acinonyx jubatus*)	Quantifying level of inbreeding and genetic diversity	[146]
Chimpanzee (*Pan troglodytes*)	Quantifying genetic diversity	[147]
Common hamster (*Cricetus cricetus*)	Consideration for breeding programs and genetic rescue	[89]
Desert bighorn sheep (*Ovis canadensis*)	Quantifying genetic diversity and disease susceptibility	[148]
Ethiopian wolf (*Canis simensis*)	Quantifying genetic diversity	[149]
Eurasian beaver (*Castor fiber*)	Quantifying genetic diversity following reintroduction	[150]
European and North American moose (*Alces alces*)	Quantifying genetic diversity	[151]
European bison (*Bison bonasus*)	Quantifying genetic diversity and pathogen resistance	[152]
European mink (*Mustela lutreola*)	Quantifying genetic diversity, genetic bottleneck, founder effect and captive breeding	[40]
European wolf (*Canis lupus lupus*)	Quantifying genetic diversity	[153]
Giant panda (*Ailuropoda melanoleuca*)	Quantifying genetic diversity and implications for the captive breeding program	[87]
Gray mouse-lemur *(Microcebus murinus*)	Quantifying genetic diversity	[154]
Hawaiian monk seal (*Monachus schauinslandi*)	Quantifying genetic variation	[155]
Iberian red deer (*Cervus elaphus hispanicus*)	Quantifying level of inbreeding and the effect of human impact	[103]
Lion-tailed macaque (*Macaca silenus*)	Quantifying genetic diversity	[156]
Malagasy mouse lemur (*Microcebus murinus*)	Quantifying genetic diversity and pathogen resistance	[56]
Malagasy giant rat (*Hypogeomys antimena*)	Quantifying genetic diversity in relation to geographic range and social system	[157,158]
Mexican wolf (*Canis lupus baileyi*)	Monitoring pathogen resistance following reintroduction	[159,160]
North American gray wolf (*Canis lupus*)	Quantifying MHC class II loci polymorphism in geographically separated regions	[161]
Northern elephant seal (*Mirounga angustirostris*)	Quantifying genetic diversity	[162]
Przewalski’s horse (*Equus ferus*)	Quantifying genetic diversity	[163]
Rhesus macaque (*Macaca mulatta*)	Monitoring intergenerational genetic changes, classifying the ancestry of research stocks	[164]
Striped mouse (*Rhabdomys pumilio*)	Quantifying genetic diversity	[165]
**Marsupials**		
Black-footed rock-wallaby (*Petrogale lateralis lateralis*)	Quantifying genetic diversity of island and mainland populations	[88]
Tammar wallaby (*Macropus eugenii*)	Quantifying level of inbreeding and disease susceptibility	[166]
Tasmanian devil (*Sarcophilus harrisii*)	Quantifying genetic diversity and understanding the development of a contagious cancer	[107,108,122]
Western barred bandicoot (*Perameles bougainville*)	Quantifying genetic diversity	[109]
